# Correction: Casein kinase 2 inhibition sensitizes medulloblastoma to temozolomide

**DOI:** 10.1038/s41388-019-1077-y

**Published:** 2019-10-28

**Authors:** Ryan T. Nitta, Sara Bolin, Emily Luo, David E. Solow-Cordero, Peyman Samghabadi, Teresa Purzner, Parvir S. Aujla, Ginikachi Nwagbo, Yoon-Jae Cho, Gordon Li

**Affiliations:** 1 Department of Neurosurgery, Stanford University, Stanford, CA USA; 2 High-Throughput Bioscience Center, Department of Chemical and Systems Biology, Stanford University, Stanford, CA USA; 3 Department of Neuropathology, Stanford University, Stanford, CA USA; 4 Department of Developmental Biology, Stanford University, Stanford, CA USA; 5 Division of Neurosurgery, University of Toronto, Toronto, ON Canada; 6 Department of Pediatrics, Papé Family Pediatric Research Institute, Knight Cancer Institute, Oregon Health & Science University, Portland, OR USA

**Keywords:** CNS cancer, Diagnostic markers

**Correction to: Oncogene**

10.1038/s41388-019-0927-y

The original version of this article contained an error in the spelling of the author David Solow-Cordero, which was incorrectly given as David Solow-Codero.

